# Human Disease Ontology 2018 update: classification, content and workflow expansion

**DOI:** 10.1093/nar/gky1032

**Published:** 2018-11-08

**Authors:** Lynn M Schriml, Elvira Mitraka, James Munro, Becky Tauber, Mike Schor, Lance Nickle, Victor Felix, Linda Jeng, Cynthia Bearer, Richard Lichenstein, Katharine Bisordi, Nicole Campion, Brooke Hyman, David Kurland, Connor Patrick Oates, Siobhan Kibbey, Poorna Sreekumar, Chris Le, Michelle Giglio, Carol Greene

**Affiliations:** 1 University of Maryland School of Medicine, Institute for Genome Sciences, Baltimore, MD, USA; 2 Dalhousie University, Halifax, NS, Canada; 3 University of Maryland School of Medicine, Baltimore, MD, USA; 4 New York University Langone Medical Center, Department of Neurosurgery, New York, NY, USA; 5 Department of Medicine, Icahn School of Medicine at Mount Sinai, New York, NY, USA

## Abstract

The Human Disease Ontology (DO) (http://www.disease-ontology.org), database has undergone significant expansion in the past three years. The DO disease classification includes specific formal semantic rules to express meaningful disease models and has expanded from a single asserted classification to include multiple-inferred mechanistic disease classifications, thus providing novel perspectives on related diseases. Expansion of disease terms, alternative anatomy, cell type and genetic disease classifications and workflow automation highlight the updates for the DO since 2015. The enhanced breadth and depth of the DO’s knowledgebase has expanded the DO’s utility for exploring the multi-etiology of human disease, thus improving the capture and communication of health-related data across biomedical databases, bioinformatics tools, genomic and cancer resources and demonstrated by a 6.6× growth in DO’s user community since 2015. The DO’s continual integration of human disease knowledge, evidenced by the more than 200 SVN/GitHub releases/revisions, since previously reported in our DO 2015 NAR paper, includes the addition of 2650 new disease terms, a 30% increase of textual definitions, and an expanding suite of disease classification hierarchies constructed through defined logical axioms.

## INTRODUCTION

The rapid growth of biomedical and clinical research in recent decades has begun to reveal novel cellular, molecular and environmental determinants of disease (1–4). However, the opportunities for discovery and the transcendence of knowledge between research groups can only be realized in conjunction with the development of rigorous, standardized bioinformatics tools. These tools should be capable of addressing specific biomedical data nomenclature and standardization challenges posed by the vast variety of biomedical data resources, such as the 3 924 249 disease-associated articles published in the past three years (1 January 2015–9 October 2018).

Standardized disease descriptors that are integrated across biomedical, clinical and genomic resources through a common language provide a human readable and machine-interpretable disease corpus. The development of a robust and well-defined ontology is critical for data sharing, effective interpretation of contextual data, rigorous computational analysis and unifying the representation of common and rare diseases. The Human Disease Ontology (DO) (http://www.disease-ontology.org), established in 2003, includes the breadth of common and rare diseases, organized as a directed acyclic graph (DAG) representing disease etiology classes, both an axiomatized OWL and an OBO formatted ontology (5). The latest DO release (GitHub, release 45, v2018-09-10) includes 9069 DOID disease terms, with 62% of terms having a textual definition. As the OBO Foundry's (www.obofoundry.org) domain ontology for human diseases, the DO semantically integrates and connected over 46 000 disease and medical vocabulary terms through extensive cross-reference mappings (MeSH, ICD, NCI’s thesaurus, SNOMED and OMIM) (6–10). The DO’s standardized descriptions of human diseases improves the capture and communication of health-related data across biomedical databases, bioinformatics tools, genomic and cancer resources.

Defining diseases based on their anatomical location has long been the standard clinical method for classification utilized in medical textbooks (11). However, as knowledge of infectious agents, clinical genetics and cellular processes grew in the past 50 years, the need arose for the classification of disease to expand to include, where known, the etiological agents of disease. The heterogeneity of genetic diseases and the multi-organ, multi-cellular origin of cancers further challenges the ontological representation of complex clinical knowledge. Alternate, inferred disease classifications provide related, unique views of related diseases and provide a novel perspective to further our understandings of commonalities of diseases located in a common anatomical location, originating from a particular cell type or resulting from a common genetic mechanism. Cancers are traditionally classified based on pathologic criteria associated with the tumor's tissue of origin. However, the identification of pathological mutations through molecular-based methods has revealed the potential of novel molecular or immunotherapy strategies for cancer treatment (12–17). Additionally, cell of origin is a promising novel mechanism for identifying molecularly related cancer types (18,19). Therefore, expanding how we define cancer molecular subtypes within a cancer molecular taxonomy is essential for advancing translational cancer research and for discerning the contribution of multiple factors towards disease initiation, progression and treatment efficacy (20–21). Although the body of knowledge regarding the cellular, genetic and environmental basis of disease continues to grow, significant gaps remain in our understanding of mechanistic pathways linking cancer subtypes to environmental triggers and underlying genetic and cellular mechanisms. Consequently, advancements in cancer genomics research are impeded. Therefore, use of semantic standards (biomedical ontologies) will address this challenge and potentially reveal novel therapeutic strategies.

## DO’s CONTENT, WORKFLOW AND DISEASE CLASSIFICATION EXPANSION

Here, we report on the significant improvements and advances to the DO database since 2015 including a broadening of the DO license, expansion of disease terms, cross reference and logical definition content, automation of data loading and quality control (QC) methods, and development of multiple, alternative disease classifications.

**Figure 1.**
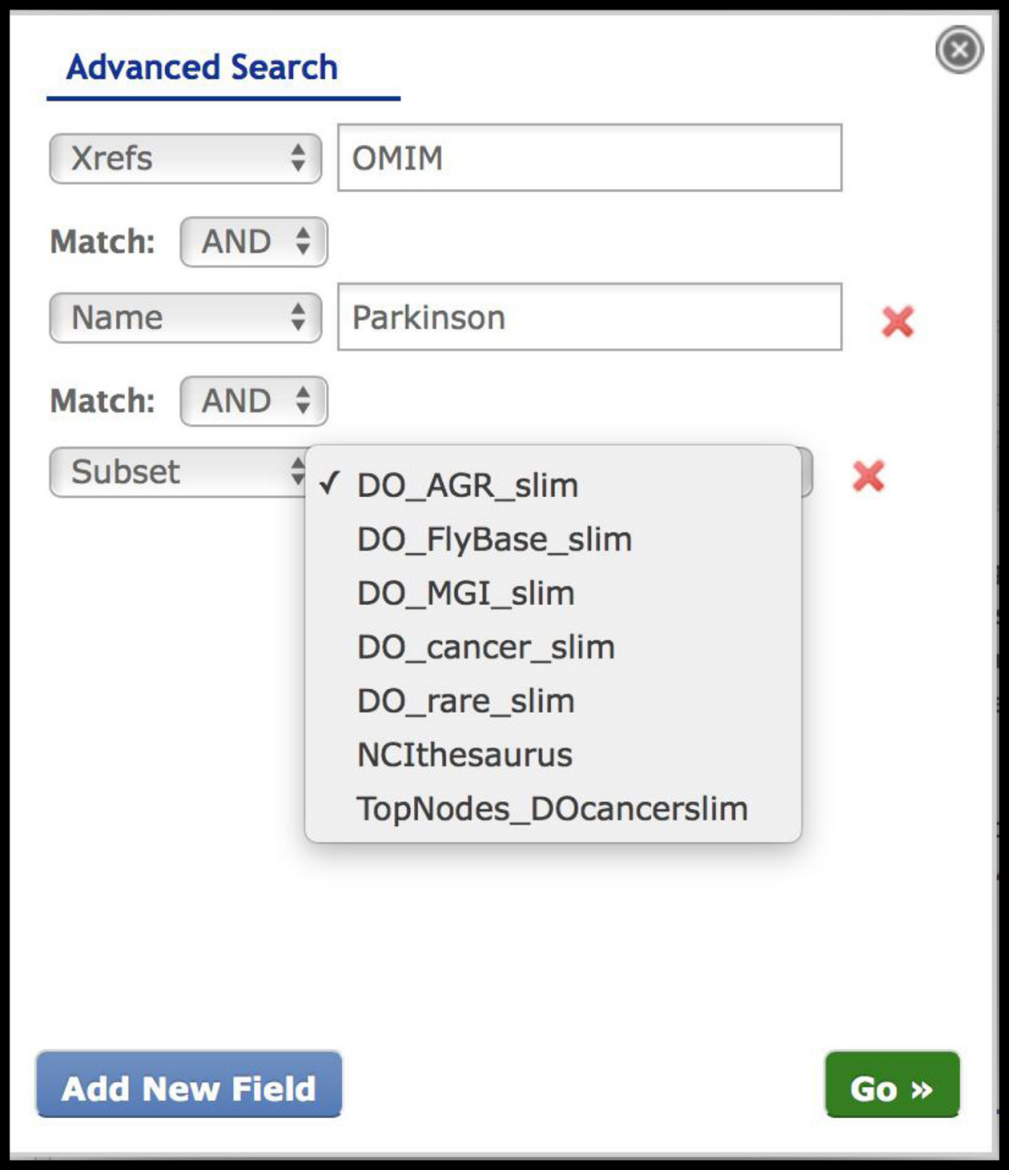
Advanced Boolean Searches. AND/OR searches of any of the DO datatypes (Name, Synonym, Definition, SubSet, DOID, Alternate ID, Xrefs) enable complex data queries of the DO Knowledgebase. For example, a search of Xrefs: OMIM, Name: Parkinson, Subset: DO_rare_slim – identifies all DO disease terms that include a cross-reference (Xref) to OMIM, where the disease name includes ‘Parkinson’ and where the disease is included in the DO_rare_slim – rare disease category. This query in DO, returns seven disease terms.

The DO website content has been maintained with periodic data updates of the DO’s regular data releases, augmented with new DO publications and update announcements via the DO News items (Home page), augmented with new Resources and Downloads content, and expanded search capabilities with the addition of Boolean Advanced searches (Figure 1), thus enabling more sophisticated querying across DO definitions, Xrefs, disease names and subsets. This query functionality enables searches that includes one or more search variables, such as the intersection of DO records that contain both OMIM and NCI thesaurus Xrefs (*N* = 738). A direct link to a DO term has been established, this query URL: http://www.disease-ontology.org/?id=DOID:12365, allows for term information to be displayed directly in the content panel, while also expanding the ontology tree to show the specific term. The DO website has seen a steady increase in usage, for example with 1.8K to 2.3K users representing 3–3.9K sessions per month (January–August 2018, Google Analytics).

### DO licensing

The DO project's data content Creative Commons licensing has been updated from CC BY 3.0 (Attribution) to CC0 (https://creativecommons.org/publicdomain/zero/1.0/) (the most open license), as of 5 April 2017, to enhance collaboration and data sharing and to encourage broad and open usage. The DO’s CC0 licensing enables the free distribution of the content of the resource thus enabling open sharing, use, and expansion (derivative works) of the content. The DO project encourages users to cite the DO project's publication, to display DOID identifiers and to link their disease terms back to the DO website. The DO was created to be a community resource, thus open content licensing is the most appropriate license for this project. Classification of human diseases is a complex endeavor, one that is best approached in an open, collaborative and community-data-driven environment.

### DO’s production environment

The DO’s production environment has moved from SourceForge/SVN to GitHub (https://github.com/DiseaseOntology/HumanDiseaseOntology/tree/master/src/ontology) (November, 2015), with day-to-day editing of DO’s editing file (doid-edit.owl) and the production of single or multi-parent OWL and OBO formatted DO ontology files (with or without inferred parents), DO subsets and customized ontology import files. As outlined in the DO’s GitHub README_DO_Files (https://github.com/DiseaseOntology/HumanDiseaseOntology/blob/master/src/ontology/README_DO_Files), the DO releases includes the production of four sets (OWL and OBO) of DO files, including custom files (created upon request). New term requests, general questions or issues are submitted to the DO team via the DO’s GitHub Issue Tracker (https://github.com/DiseaseOntology/HumanDiseaseOntology/issues). Since moving to GitHub, the DO team has received over 500 GitHub requests for new disease terms, modification or expansion of DO terms, suggestions, topics for review, specific SPARQL queries (e.g. DO terms for all mental health disorders) and assistance with research projects. Resolution of open tickets is an ongoing daily activity of the DO project.

### DO files & term counts

**Figure 2.**
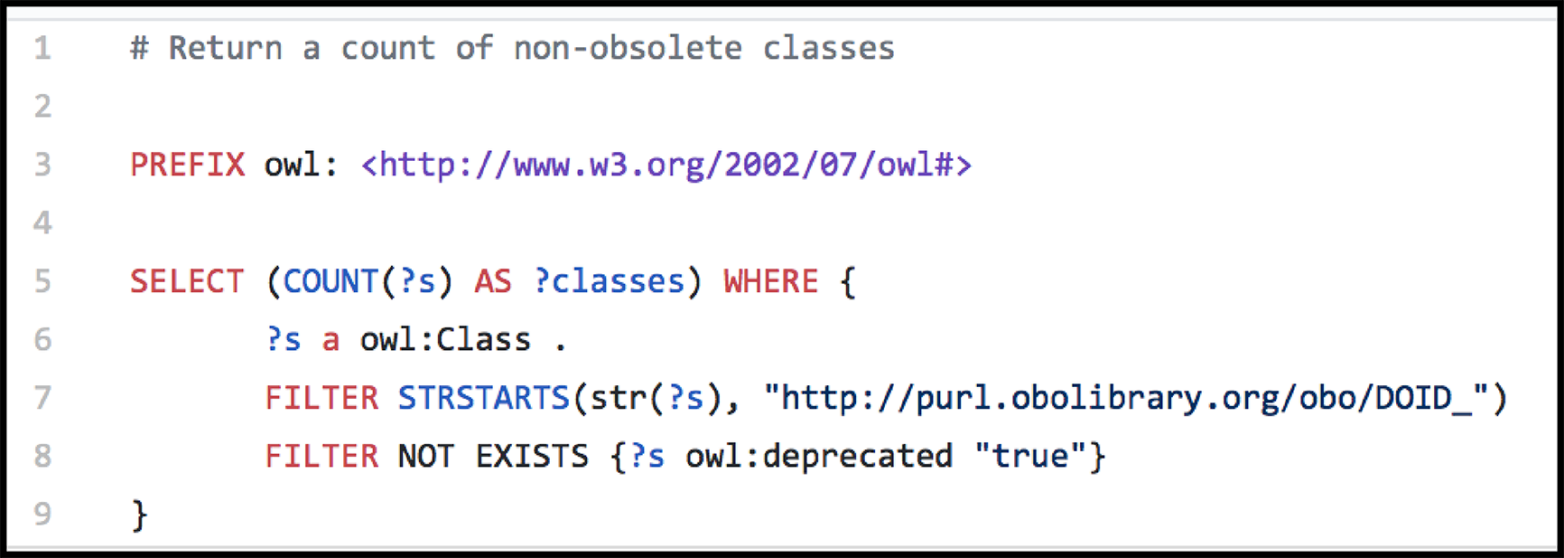
An example DO SPARQL: doid-report.rq.

Class counts differs across the DO’s release files, depending on whether the file include obsolete DO terms and the imported ontology terms, as outlined in Table 1. The DO’s GitHub repository includes additional resources, such as a set of SPARQL queries (Figure 2) utilized for assessing (QC) the content and quality of the files produced. Additionally, the SPARQL queries are provided to enable DO users to explore DO content in a novel way.

**Table 1.** DO file names and content, class counts

File name (.owl and .obo)	Content	Hierarchy	Classes	Total # classes
doid	OBO Foundry format	asserted is_a with, Equivalent To Axioms, SubClass Of Statements	imports, DOIDs	17,579
doid-non-classified	DO file format	asserted is_a	DOIDs: non-obsolete (9,069)	11,463
doid-merged	MGI custom file	asserted is_a, inferred parents	imports, DOIDs, omim_susceptibility	15,183

Note: The file, doid-non-classified, is an equivalent file (in content and structure) to HumanDO.obo. The DO project has continued to produce the HumanDO files, as this was the original naming convention used by the project for ∼10 years and was included in several publications. Total class counts include non-obsolete and obsolete DOIDs (DO identifiers). OBO Foundry (http://www.obofoundry.org) (22). For DO’s GitHub release # 45, there are 9,069 DOIDs, also retrievable from the DO’s website.

The DO’s growing library of SPARQL code, verification (QC) and reporting (metrics) includes:

  QC: dnc-verity-connectivity.rq: Ensure all classes are a subclass of disease

   dnc-verify-single-parent.rq: Ensure that classes have exactly one parent

   verify-no-orphans.rq: Ensure that no class is orphaned (excluding top level ‘disease’)

 reporting: xref-report.rq: Return a count of Xrefs on non-obsolete classes

   import-report.rq: Return a count of each import

   logical-report.rq: Return a count of logical definitions

   **doid-report.rq: # Return a count of non-obsolete classes**

   def-port.rq: # Return a count of classes with definitions

   DO_no_defs.rq: identify set of DO terms needing a textual definition

A set of 12 customized import files, that represent subsets of other OBO Foundry ontologies are produced with each release (https://github.com/DiseaseOntology/HumanDiseaseOntology/tree/master/src/ontology/imports) along with OWL and OBO formatted DO subset files (slims) that represent a selected set of DO terms as a smaller version of the entire DO file. For the subsets, the OBO versions only contain DO terms, the OWL versions also contain the associated logical axioms (https://github.com/DiseaseOntology/HumanDiseaseOntology/tree/master/src/ontology/subsets). These customized DO slim files include: AGR (Alliance for Genome Resources) (https://www.alliancegenome.org), FlyBase (23), MGI, cancer, rare disease and NCI thesaurus, are used to annotate cancer variants, animal models and human genes to human diseases, rather than using the full DO. The NCI thesaurus subset was expanded in August 2018 to include all NCI thesaurus terms in the DO (4302 terms).

### DO content update

#### Term expansion

**Figure 3.**
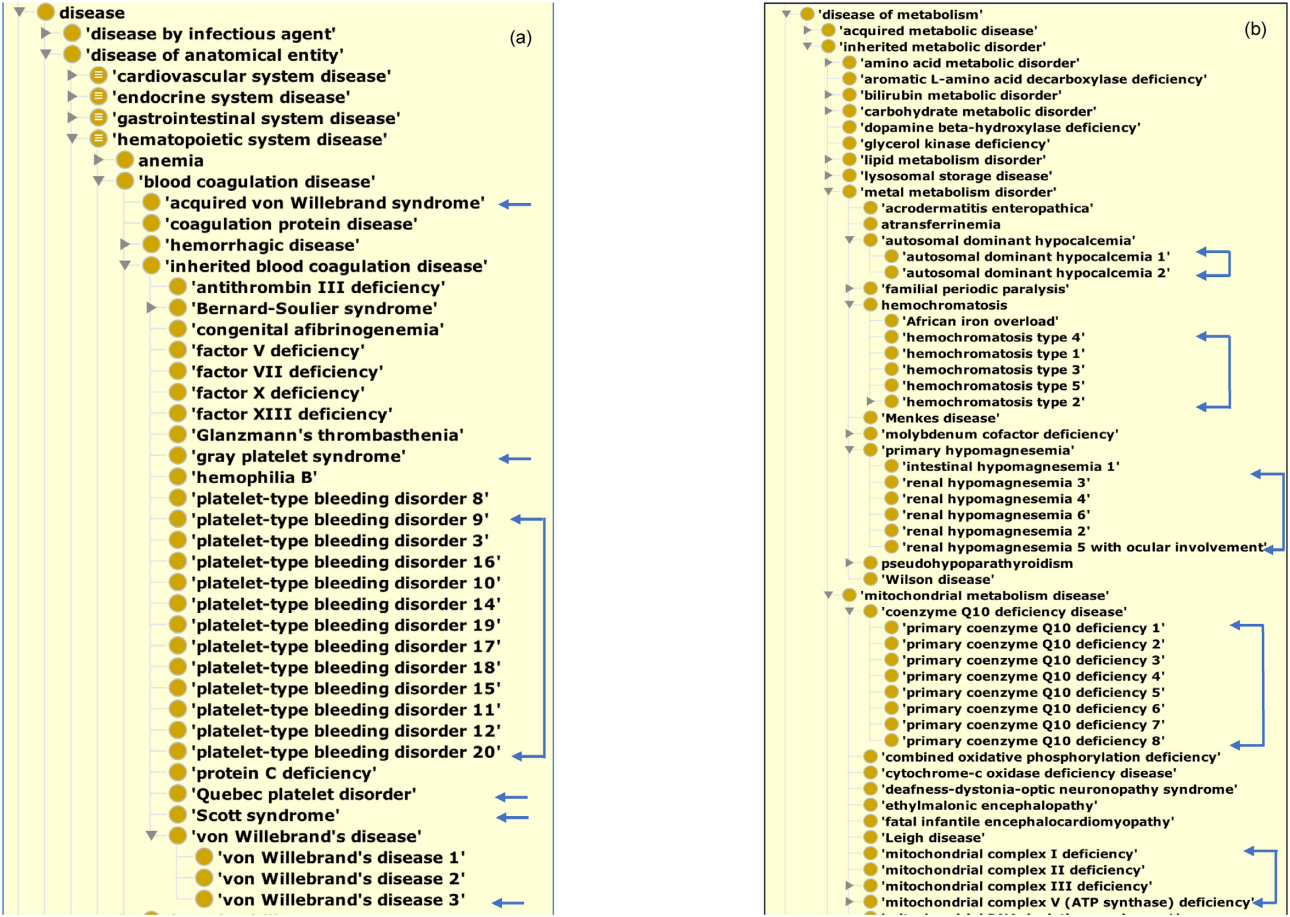
Example areas of term expansion: (**A**) hematopoietic system diseases and (**B**) inherited metabolic disorders. Blue arrow indicates new DO terms.

The latest release of the DO [release 45; GitHub release v2018-09-10], with 9069 ‘non-obsolete’ classes (disease terms), represents an increase of 2650 disease terms, with 62% (5642/9069) of DO terms defined with a textual definition, since 2015. The new disease terms have been added across the DO, in particular expanding the structure of DO’s hematopoietic, neurodegenerative, inherited metabolic disorders and genetic diseases (Figure 3). Human disease knowledge is continually integrated into the DO, as evidenced by the 190 SourceForge revisions (from revision 2702 on 6 October 2014 to revision 2895 on 11 March 2016) and 45 GitHub releases (March 2016–September 2018) since 2015. The legacy DO SourceForge repository will be retired by the end of 2018.

#### Automated workflows

Various parts of the DO workflow have been automated using the ROBOT command line tool (http://robot.obolibrary.org). ROBOT, developed in Java and available open source on GitHub (https://github.com/ontodev/robot), has also been implemented in other OBO Foundry ontologies such as the Ontology for Biomedical Investigations (24), the Gene Ontology (25), the Evidence and Conclusion (ECO) ontology (26) and the Immune Epitope Database's (IEDB) (27) MHC Restriction Ontology (28, http://ceur-ws.org/Vol-1515/demo6.pdf). ROBOT allows developers to quickly integrate logical definitions and class annotations through the ‘template’ command, which transforms a spreadsheet into OWL axioms. New content is added to the DO from structured ROBOT template spreadsheets that include rows of new content with columns for the DOID, disease name, definition, definition Xref, parent DOID, synonyms and logical axioms. The ROBOT template spreadsheet is organized by the first two rows, to specify the ROBOT terms (e.g. Label) and corresponding ontology terms (e.g. A rdfs:label) for the disease name. Introducing ROBOT templates to the DO production pipeline has enabled the DO team and collaborators (such as Mouse Genome Informatics (MGI) (29,30), Rat Genome Database (RGD) (31), IEDB and Wikidata (32) to coordinate data updates and additions through shared Google spreadsheets. Following thorough quality control and validation, the new axioms are programmatically added to DO, thus greatly reducing the time for data integration and improving overall data quality.

Some of the content that has been recently added to DO using ROBOT templates includes:

- rare cancers from Clinical Interpretation of Variants in Cancer (CIViC) (33), this work has enhanced DO’s content, for clinically actionable cancer variants, as represented in literature, and defined in the CIViC database.
- 140 new allergy diseases from IEDB and related logical definitions linking classes to UBERON (anatomy), FoodOn (food allergic triggers), ChEBI (chemical allergic triggers), and NCBITaxon (34–37).
- 43 new human diseases from MGI complete with cross references, synonyms, textual definitions, and parent classes.
- 50 OMIM, 1491 GARD (rare disease) (38) and 655 MeSH cross references identified by Wikidata users, extracted from Wikidata database and validated by the DO team.
- 1433 textual definitions added to existing DO classes to enrich the DO’s cancer classification, constructed by seven second to fourth year University of Maryland School of Medicine medical students.
- OMIM splits: new DO terms for genetic disease subtypes: curated by MGI—(469 new DO terms) and RGD (201 new DO terms). This is an ongoing, weekly activity as new terms are created in OMIM, curated by MGI, RGD and DO, then added to DO. This work has resulted in a vast expansion of genetic diseases, and their associated OMIM IDs in the DO, contributing significantly to the DO’s ongoing focus with MGI and RGD to represent OMIM’s Phenotypic series (90 OMIM:PS# in DO, and 3273 OMIM ID Xrefs) as disease subtypes.

Adding new subtypes to the DO, as defined by the OMIM Phenotypic Series, is an ongoing curation task for the DO team. The DO parent term, e.g. primary hypomagnesemia [DOID:0060879] is annotated with the OMIM Phenotype Series ID (e.g. PS602014), if it has been defined by OMIM. The set of OMIM phenotypes define the new DO terms, one OMIM ID per DO term. As the Phenotypic Series expand, the DO team adds the new DO terms as needed. Given the breadth of the Phenotypic Series and their ongoing development, there can be a time lag between the addition of terms. MGI identifies each week the creation of new OMIM phenotypes, additions to Phenotypic Series or updates to OMIM IDs and coordinates these changes in the DO OMIM curation queue that we jointly maintain.

ROBOT has also been implemented to automate the entire DO release process. A series of commands are contained in a ‘Makefile’ which automatically builds target products (DO’s production OWL and OBO files, slims, subsets) in under 10 minutes. This process includes a series of customizable ontology error and warning checks (e.g. white spaces, duplicate synonym, multiple asserted superclasses, invalid Xref, missing obsolete label) produced from the ROBOT ‘report’ command, which identifies areas of improvement for the ontology file as curation QC output files. The ROBOT release process has also integrated a series of time-intensive, manual file checks (e.g. dates, versioning). Additionally, the ROBOT tool's commands are utilized to update import ontologies, remove and filter parts of the DO to create subsets, and annotate final release files.

### To etiology and beyond—disease classifications

**Figure 4.**
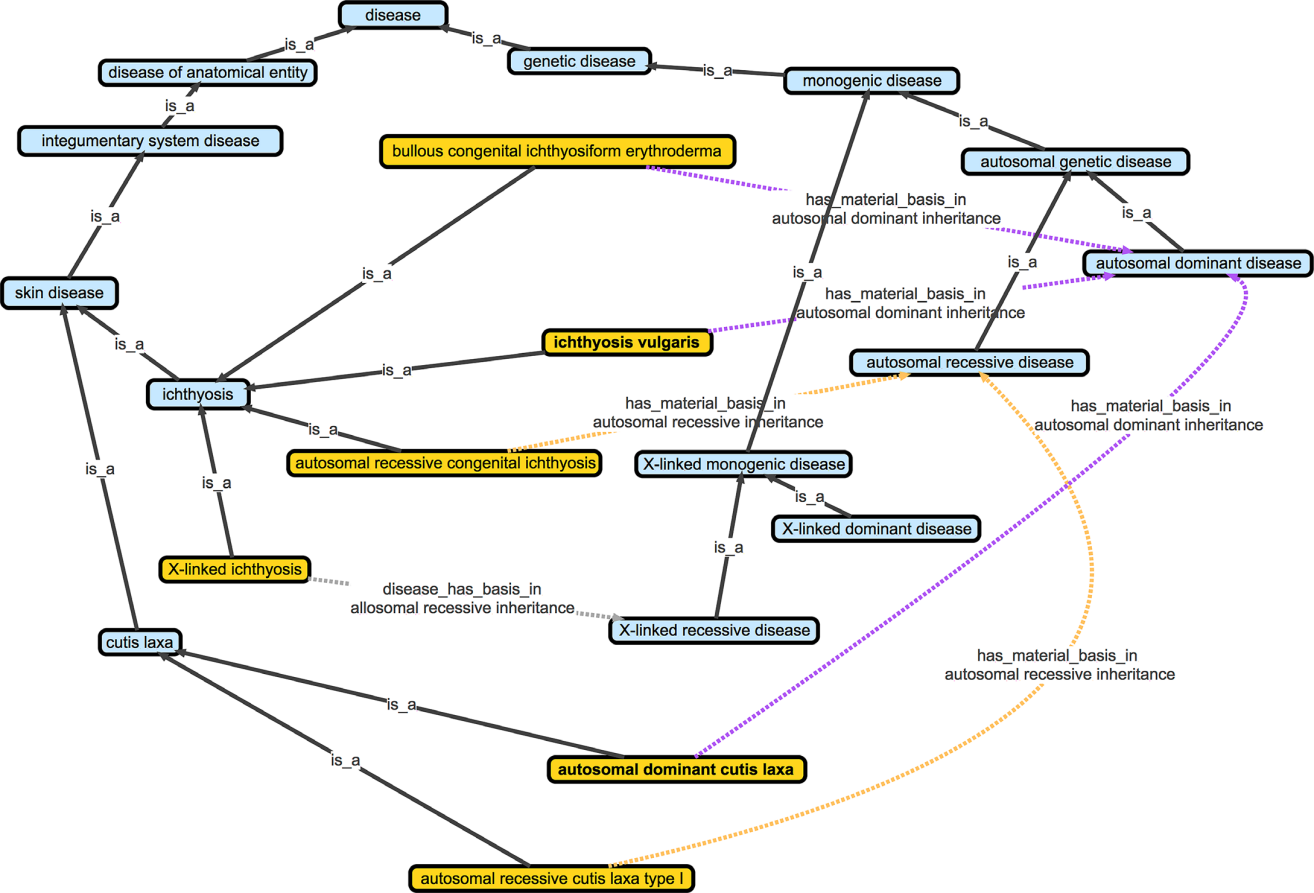
Skin disease logical axioms, define inferred disease parents. Integration of a SubClass Of logical axiom for ‘ichthyosis vulgaris’ and ‘autosomal dominant cutis laxa’ [BOLD] (‘has material basis in’ some ‘autosomal dominant inheritance’) and an ‘Equivalent To logical axiom for ‘autosomal dominant disease’ (disease and (‘has material basis in’ some ‘autosomal dominant inheritance’)), where ‘autosomal dominant inheritance is from the Genotype Ontology (http://www.obofoundry.org/ontology/geno.html) creates ‘inferred’ child to parent DO relationships, thus both skin diseases are defined as inferred child terms of DO’s ‘autosomal dominant disease’.

The DO’s classification expansion is evolving through a structured, step-wise approach with the goal of representing DO diseases through multiple-inferred classifications. The classifications are encoded within the DO’s OWL files, defined by Equivalent To axioms and SubClass Of statements, queryable and viewable at the EBI’s Ontology Lookup Service (39), https://www.ebi.ac.uk/ols/ontologies/doid. To date the DO contains 730 Equivalent To axioms and 3612 SubClass Of statements. The eleven types of axioms (e.g. anatomical, genetic, cell type), being constructed in the DO are defined by a specific RO (Relation Ontology) (40) term and an OBO Foundry term. For example, a sequence variant axiom could be constructed as ‘has_material_basis_in some (loss_of_function_variant and maternal_uniparental_disomy). The associated OBO Foundry imports have been customized to include the subset of their terms utilized by the DO for defining axioms, thus reducing the overall size of the DO’s OWL files. When a new OBO ontology term is needed, the ROBOT tool is utilized to augment the DO’s imports. Expansion of DO’s inferred classifications allows users to examine related diseases from multiple perspectives. For example, logical definitions for two skin diseases, ichthyosis and autosomal dominant cutis laxa (Figure 4), demonstrates.

To date, logical axioms for the DO’s inferred-anatomy classification are complete, DO’s inferred-cell type and inferred-genetic classifications are in progress. To rigorously build the DO inferred-classifications, the follow protocol has been established, with DO’s inferred-anatomy classification as an example: scripted identification of anatomy terms (e.g. heart, pericardium, artery, vein, cardiac, aortic) utilized for each branch of DO’s ‘disease of anatomical entity’ branch to:

1. define Equivalent To axioms for parent terms, for example, for the ‘cardiovascular system disease’ branch, Equivalent To: ‘disease and located_in some cardiovascular system’;
2. search the entirety of DO to identify other diseases that were likewise located_in one of the anatomical locations (outside of the ‘disease of anatomical entity’);
3. define SubClass Of statements for disease terms we want to define as an inferred child of ‘cardiovascular system disease’, such as ‘autoimmune cardiomyopathy’ with the SubClass Of statement: ‘located_in some heart’;
4. The DO has expanded anatomical mechanistic models for DO’s 12 ‘disease of anatomical entity’ branches and 14 organ system cancer branches and is in the process of defining cell of origin models for DO’s 11 cell type cancer branches by defining the most granular anatomical location and cell of origin SubClass Of statements for each cancer type using the ‘located_in’ and ‘has_material_basis_in’ relationships.

Further models will be defined for DO’s pre-malignant neoplasm and benign neoplasm terms. The tissue and cell of origin axioms (logical definitions) enable querying and enhanced views of the DO with logical definitions associated with OBO Foundry ontologies), UBERON (anatomy) and the Cell Ontology (cell types) (41). The expansion DO’s mechanistic classifications for DO’s organ system and cell type cancers are producing multi-mechanistic cancer models. These novel models of cancer will provide a robust backbone for complex cancer queries, exposing a multi-faceted, cancer classification systems in an intuitive format with data managed within a rigorous semantic structure.

### DO user community expansion & metrics

The DO user community has continued to expand over the past three years, in addition to adoption of the DO by the Model Organism Databases (MOD), DO has been integrated into the Alliance for Genome Resources, thus facilitating gene and allele comparative analysis. For example, the AGR’s query interface presents 34 alleles associated with neurodegenerative diseases (https://www.alliancegenome.org/search?category= allele&diseaseDocuments.name=neurodegenerative%20disease&q=syndrome%5B1%5D%20%28Dme%29). Whereas, a query on a specific disease, e.g. Huntington's disease, returns the 128 AGR gene associations (https://www.alliancegenome.org/disease/DOID:12858#associations) from RGD, MGI, ZFIN (42), FlyBase and WormBase (43).

Determining usage of an open-source biomedical resources, such as the DO, is a non-trivial activity, involving direct project citations, mentions of the resource, project URL or unique identifier (e.g. DOID) in PubMed and Google searches. Utilizing this multi-factored approach (on a monthly basis), the DO team has identified a body of 336 DO project citations (as of August 2018), an increase from ∼50 citations in 2015. This set of citations has been compiled as a public PubMed MyNCBI collection (DO_citing_papers: https://www.ncbi.nlm.nih.gov/sites/myncbi/lynn.schriml.1/collections/49204559/public/). This MyNCBI collection represents the growing number of instances of integration of DO in databases, research studies, bioinformatics tools.

## FUTURE DIRECTIONS

In the near term, the DO’s website will be getting a face lift, to enhance educational materials, connect to a broader user community, and improve coordination of outreach opportunities. The DO’s website v2.0 is under construction to provide querying of the DO’s inferred classifications (doid.owl file). Keep an eye on the DO’s growing number of inferred-classifications for organ and cell type cancers and genetic diseases, which will be updated with DO’s monthly GitHub releases. The first set of inferred disease classifications will be expanded to include inferred disease classification hierarchies for genetic, inheritance, symptom, phenotype, transmission method and pathogenic agent. For example, observable characteristics or traits (phenotypic traits) from the Human Phenotype Ontology (HPO) (44) will define logical axioms with the ‘has_phenotype’ relation for phenotypes of syndromes and genetic diseases. A complex etiology-based disease classification is in the early stages of development, for an early preview, query Prader-Willi syndrome at EBI’s Ontology Lookup Service. The DO will continue to grow in content and scope, integrating additional rare diseases, newly published diseases and revising etiology classifications as knowledge evolves. The DO’s team has expanded substantially in the past year. A near term goal for the team is to address the current backlog of GitHub tickets.

## DATA AVAILABILITY

The Human Disease Ontology content is available, under Creative Commons CC0 (CC0 1.0 Universal) license, in the GitHub repository (https://github.com/DiseaseOntology/HumanDiseaseOntology/tree/master/src/ontology), at the OBO Foundry (http://www.obofoundry.org/ontology/doid.html, http://purl.obolibrary.org/obo/doid.owl), from the DO’s website (http://www.disease-ontology.org), and the Ontology Lookup Service (OLS) at EBI (https://www.ebi.ac.uk/ols/ontologies/doid).
